# Gender differences of morphological and hemodynamic characteristics of abdominal aortic aneurysm

**DOI:** 10.1186/s13293-020-00318-3

**Published:** 2020-07-21

**Authors:** Zujie Gao, Jiang Xiong, Zengsheng Chen, Xiaoyan Deng, Zaipin Xu, Anqiang Sun, Yubo Fan

**Affiliations:** 1 School of Biological Science and Medical Engineering, Beijing Advanced Innovation Center for Biomedical Engineering, Key Laboratory for Biomechanics and Mechanobiology of Ministry of Education, Beihang University, Beijing, 100083 China; 2 Department of Vascular and Endovascular Surgery, The Chinese PLA General Hospital, Beijing, 100853 China; 3 College of Animal Science, Guizhou University, Guiyang, 550025 China; 4 Key Laboratory of Rehabilitation Aids Technology and System of the Ministry of Civil Affairs, National Research Center for Rehabilitation Technical Aids, No. 1 Ronghuazhong Road, Beijing BDA, Beijing, 100176 China

**Keywords:** Gender difference, Abdominal aortic aneurysm, Hemodynamics, Morphological

## Abstract

**Background:**

Gender difference in cardiovascular diseases (CVDs) is an important topic in the field of cardiovascular medicine. In this study, we focused on the mortality difference of abdominal aortic aneurysms (AAA), which is higher for female than that of male. The aim of this study was to verify whether morphological and hemodynamic factors play their roles in this phenomenon.

**Methods:**

Patient-specific AAA models of 11 females and 23 males with similar age and body mass index (BMI) have been reconstructed based on clinical computed tomography (CT) data. Firstly, the morphological parameters (diameters, curvature, intraluminal thrombus volume, etc.) of AAA models and lumbar vertebrae models were collected and analyzed. Then, based on statistical results of morphological parameters, uniformed male and female AAA models were reconstructed, and hemodynamic simulations were conducted respectively. In post-processing, the hemodynamic performances induced by gender-different morphological geometries were analyzed and compared.

**Results:**

The comparison of morphological parameters revealed that the average curvature of lumbar vertebrae and AAA centerline of female AAA models were obviously higher than that of the male. The amount of intraluminal thrombus in female AAA models was relatively lower than that of the male. According to the hemodynamic simulation, the uniform female AAA model has higher peak pressure, lower oscillatory shear stress index (OSI), and lower relative residence time (RRT) than that of the male model, all of which put female AAA to a relatively higher risk hemodynamic situation.

**Conclusions:**

The morphological and hemodynamic features of AAA have very obvious gender differences that would induce higher risk of rupture for female AAA biomechanically. These findings would help to explore the mechanism of gender differences in AAA and draw attention to gender-specific consideration for AAA treatment. More morphological and hemodynamic indictors are suggested to be involved in the future guidelines.

## Background

Gender differences of cardiovascular diseases (CVDs) between female and male have been reported and discussed by recent literatures [1]. Abdominal aortic aneurysm (AAA) is one of the typical gender-related aorta diseases. AAA is a degenerative disease manifested by an irreversible enlargement in the abdominal aorta which has been regarded as one of the most threatening diseases for elder people (> 65 years old) [2]. Rupture is a dangerous event for AAA, with a mortality rate exceeding 80% [3]. The rupture rate of AAA in female is more than 4 times that of male [4–6].

Although the gender difference of AAA has been validated by multiple statistical meta-analysis [7], its mechanism is still unclear and has not been considered in most current surgical decision-making guidelines. The ignorance of gender differences in the clinical procedure would cause an unpredictable influence on the survival rate and quality of life of AAA patients. Understanding AAA gender differences would help make more accurate surgical decisions for patients.

Previous studies have found that hemodynamic factors play a significant role in the growth and rupture of AAA [8]. Blood pressure, peak wall stress (PWS), oscillatory of flow velocity, etc. have been confirmed as key factors affecting AAA physiology and pathology [9, 10]. Meanwhile, various studies have shown that the gender difference in the risk of AAA rupture might be caused not only by physiological factors (such as LDL and sex hormone) [11–13] but also by anatomical factors [14] (such as the body size and the diameter of the aorta [15]). Therefore, the present study hypothesized that the differences in anatomical structure between female and male would influence the hemodynamics in the AAA which might induce different biological consequences.

In the present study, in order to further explore the mechanism of AAA gender differences, the morphology and hemodynamic differences between male and female were investigated, and some morphological and hemodynamic indictors have been found that may be related to the AAA gender differences.

## Methods

### Patients

**Fig. 1:**
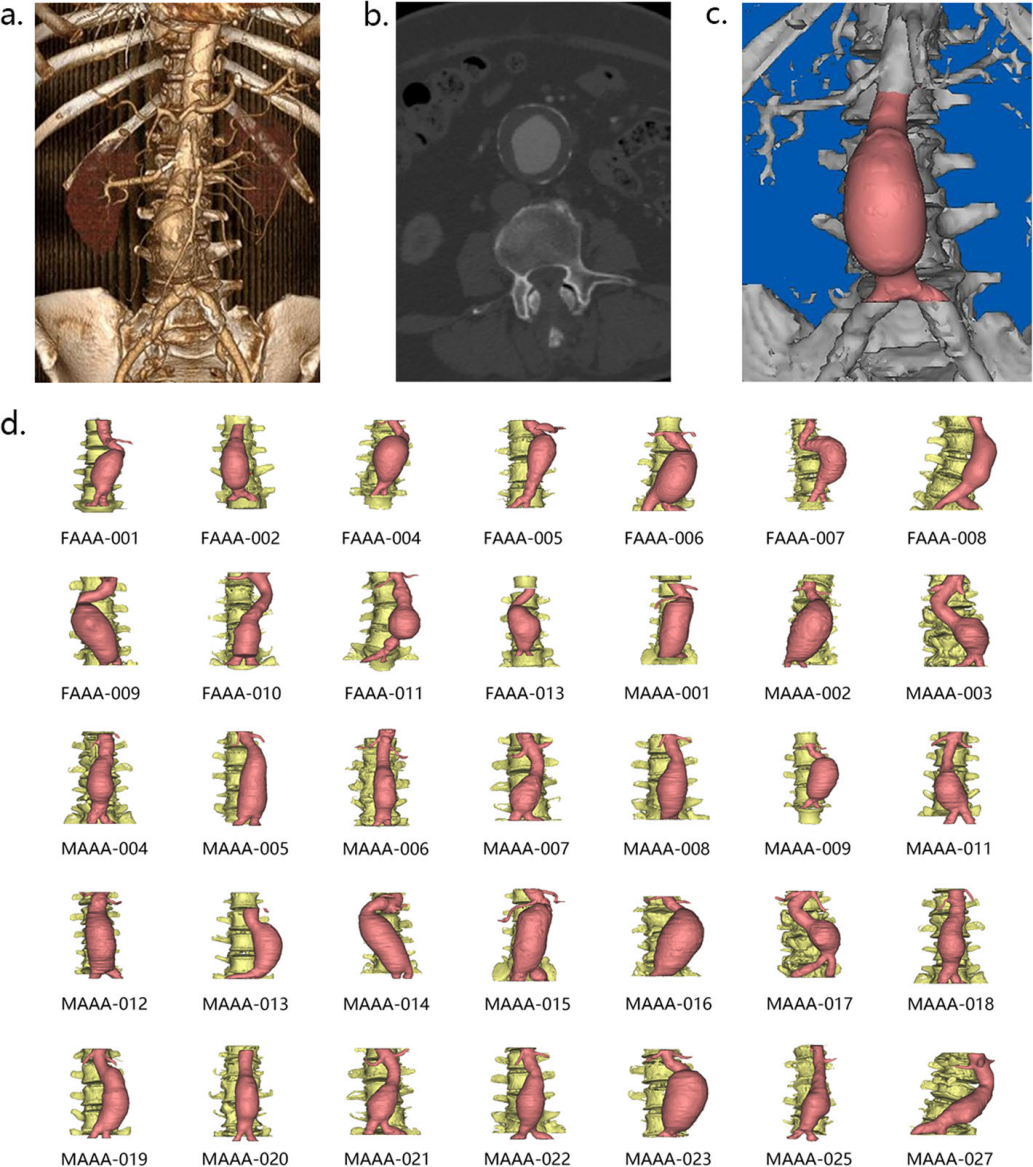
Segmentation and 3D reconstruction of AAA. **a** Abdominal computed tomography (CT) imaging, supported by the RadiAnt software. **b** Segmentation of lumbar vertebrae, blood, and blood vessels by grayscale and anatomy (cross section). **c** Reconstructed abdominal aorta (shown in pink) in the Mimics software. **d** All AAA models used in this study.

In total, 11 female and 23 male patients diagnosed with AAA by computed tomography (CT) scans (shown in Fig. 1a) in the Chinese PLA General Hospital between 2017 and 2018 were randomly selected. Patient demographic information (age, weight, height, blood pressure, and the heart rate), AAA risk factors (cardiovascular disease (such as coronary artery heart disease and cerebral infarction), hypertension, diabetes, smoking history, and chronic obstructive pulmonary diseases (COPD)), and biochemical information (hemoglobin, total cholesterol, triglycerides, high-density lipoprotein cholesterol, and low-density lipoprotein cholesterol) are shown in Table 1. Body mass index (BMI) and body surface area (BSA) were calculated by height and weight, where the BMI was defined as [15]

1 $$ \mathrm{BMI}=\frac{\mathrm{weight}\left(\mathrm{kg}\right)}{{\mathrm{height}}^2\left({\mathrm{m}}^2\right)} $$

**Table 1:** Clinical data of all patients

Gender	Female (*n* = 11)	Male (*n* = 23)	*p*
Age (year)	72.6 ± 8.2	72.9 ± 3.6	0.901
BMI (kg ∙ m^−2^)	24.9 ± 4.01	23.7 ± 2.23	0.278
BSA (m^2^)	1.65 ± 0.136	1.81 ± 0.129	0.0025
Systolic (mmHg)	145.2 ± 15.4	137.3 ± 13.4	0.131
Diastolic (mmHg)	76.3 ± 9.9	79.9 ± 11.9	0.321
Heart rate (bpm)	81.4 ± 11.5	78.9 ± 13.3	0.571
Cardiovascular diseases	15.40%	40.70%	0.193
Hypertension	69.20%	51.90%	0.310
Diabetes	38.50%	11.10%	0.0440
Smoking	15.40%	92.60%	5.4E−09
COPD	7.70%	3.70%	0.599
Hemoglobin (g/L)	122.7 ± 13.6	130.0 ± 16.9	0.628
TC (mmol/L)	4.84 ± 0.92	4.47 ± 1.29	0.332
TG (mmol/L)	1.78 ± 1.54	1.23 ± 0.74	0.255
HDL-C (mmol/L)	1.14 ± 0.31	1.14 ± 0.31	0.9997
LDL-C (mmol/L)	3.11 ± 0.83	2.88 ± 1.12	0.484

*COPD* chronic obstructive pulmonary diseases, *TC* total cholesterol, *TG* triglyceride, *HDL-C* high density lipoprotein cholesterol, *LDL-C* low density lipoprotein cholesterol

BSA was defined as [16]

2 $$ \mathrm{BSA}=0.20247\times \left[\mathrm{weight}{\left(\mathrm{kg}\right)}^{0.425}\times \mathrm{height}{\left(\mathrm{m}\right)}^{0.725}\right] $$

There is no difference in average age (72.6 vs 72.9 years; *p* = 0.901) or average BMI (24.9 vs 23.7 kg ∙ m^−2^; *p* = 0.278) between females and males (Table 1). Systolic pressure, diastolic pressure, and heart rate were the same for females and males. Some common complications of AAA, such as cardiovascular disease, hypertension, and COPD, are not significantly different between genders. Diabetes which is considered a protective factor for AAA [17] is more common among females (38.5% vs 11.15%; *p* = 0.044). Clinical details are also summarized in Table 1.

### AAA model reconstruction

The CT images were obtained via a dual-source CT scanner (SOMATOM Definition Flash, SIEMENS, Germany) with injection of 70~90 ml of contrast agent with 50 ml of saline chaser, threshold 80 HU; rotation speed 500 ms; collimation 64; slice 1.0 mm; pitch 1.0; voltage 100 kV; current 200~350 mA; image resolution 512 × 512; and slice increment 0.699 mm. The CT data were imported into the Mimics software (v9.0, Materialise, Ann Arbor, MI, Belgium) for segmentation, and the abdominal aorta, lumbar vertebra, and blood were identified by the grayscale division shown in Fig. 1b. The reconstructed AAA models and lumbar vertebrae models are shown in Fig. 1c. All the reconstructed AAA models are shown in Fig. 1d.

### Morphological parameter extraction

**Fig. 2:**
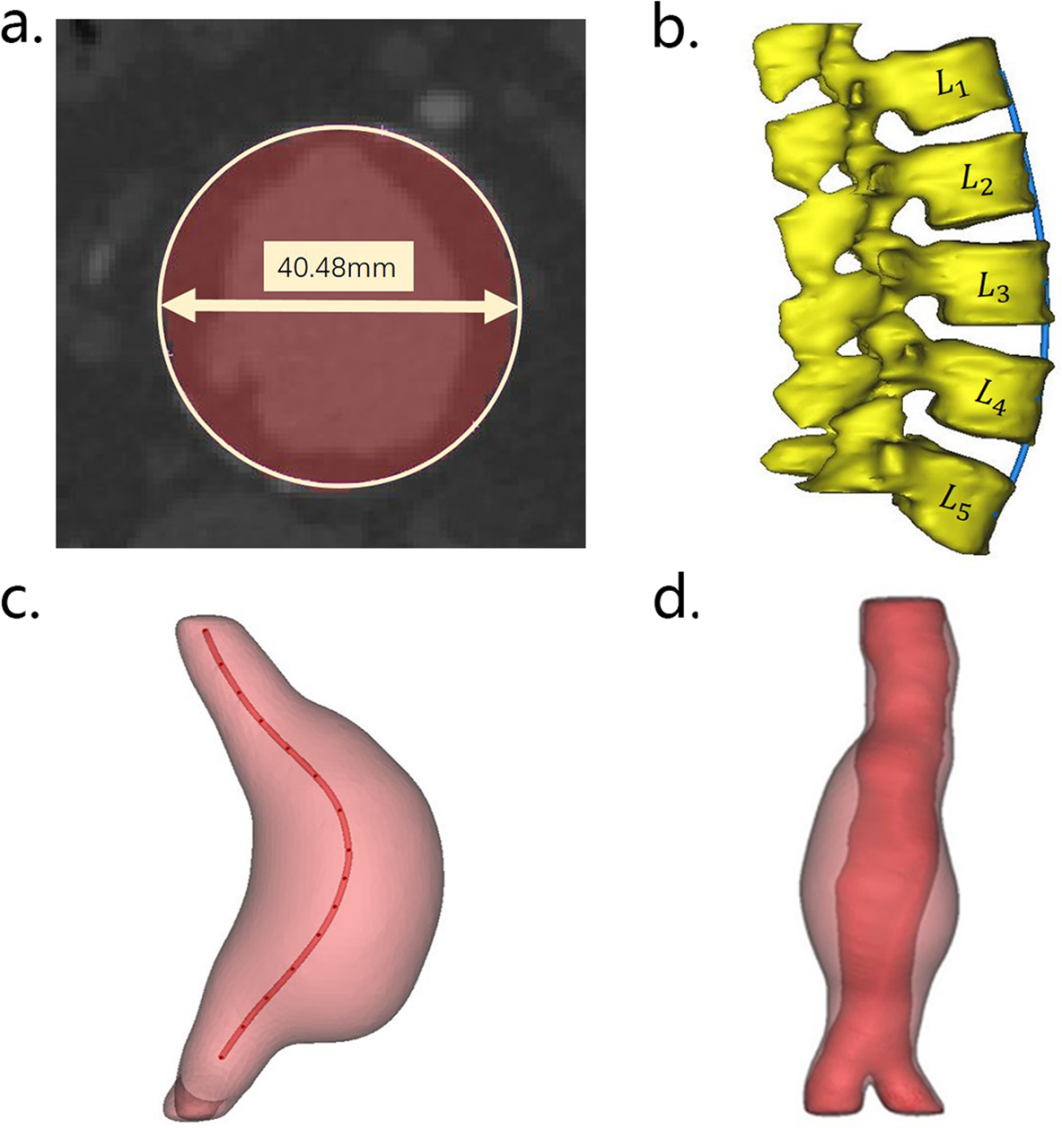
Morphological feature parameters of AAA. **a** Diameter measurement. **b** Obtainment of lumbar vertebrae curve. Then, lumbar vertebrae curve was obtained by connecting the midpoint of the five lumbar vertebrae shown in dark blue. **c** Centerline acquisition. The centerline is in dark red. **d** Calculation of ILT percentage volume. The total volume of AAA is shown in pink and the volume of blood is shown in dark red

Based on the models reconstructed above, the morphological parameters were measured, as shown in Fig. 2, including the diameters of the AAA (Fig. 2a), the lumbar vertebrae curve (Fig. 2b), the centerline of AAA (Fig. 2c), and the thrombus volume (Fig. 2d). The aneurysm length was measured axially from the renal artery bifurcation to the aortic bifurcation.

According to the diameter information, two indicators which are commonly used to measure the risk of rupture were calculated. One is defined as the ratio of the maximum diameter to the diameter of the proximal normal aorta [18, 19] expressed as the expansion factor:

3 $$ \mathrm{Expansion}\ \mathrm{factor}=\frac{\mathrm{the}\ \mathrm{maximum}\ \mathrm{diameter}\ \mathrm{of}\ \mathrm{AAA}}{\mathrm{proximal}\ \mathrm{normal}\ \mathrm{aorta}\ \mathrm{diameter}} $$

The other one is aortic size index (ASI) [20] defined as

4 $$ \mathrm{ASI}=\frac{\mathrm{aneurysm}\ \mathrm{diameter}\left(\mathrm{cm}\right)}{\mathrm{BSA}\left({\mathrm{m}}^2\right)} $$

The lumbar vertebrae curve was obtained by connecting the anterior midpoint of the five lumbar vertebrae (L_1_~L_5_) in dark blue, as shown in Fig. 2b. The centerline was calculated by the medCAD function of the Mimics software with a smooth factor of 0.5 shown in Fig. 2c as dark red line. Based on the above curves, the projection of the curve on the sagittal plane was obtained, and the curvature was calculated.

The total volume of abdominal aortic aneurysm (*V*_a_) and the volume of blood (*V*_b_) were used to calculate intraluminal thrombus (ILT) percentage volume. The calculation formula is as follows:

5 $$ \mathrm{ILT}\ \mathrm{percentage}\ \mathrm{volume}=\frac{\left({V}_{\mathrm{a}}\right)-\left({V}_{\mathrm{b}}\right)}{\left({V}_{\mathrm{a}}\right)} $$

In Fig. 2d, *V*_a_ is shown in pink, and *V*_b_ is shown in dark red.

### Uniform model generation

**Fig. 3:**
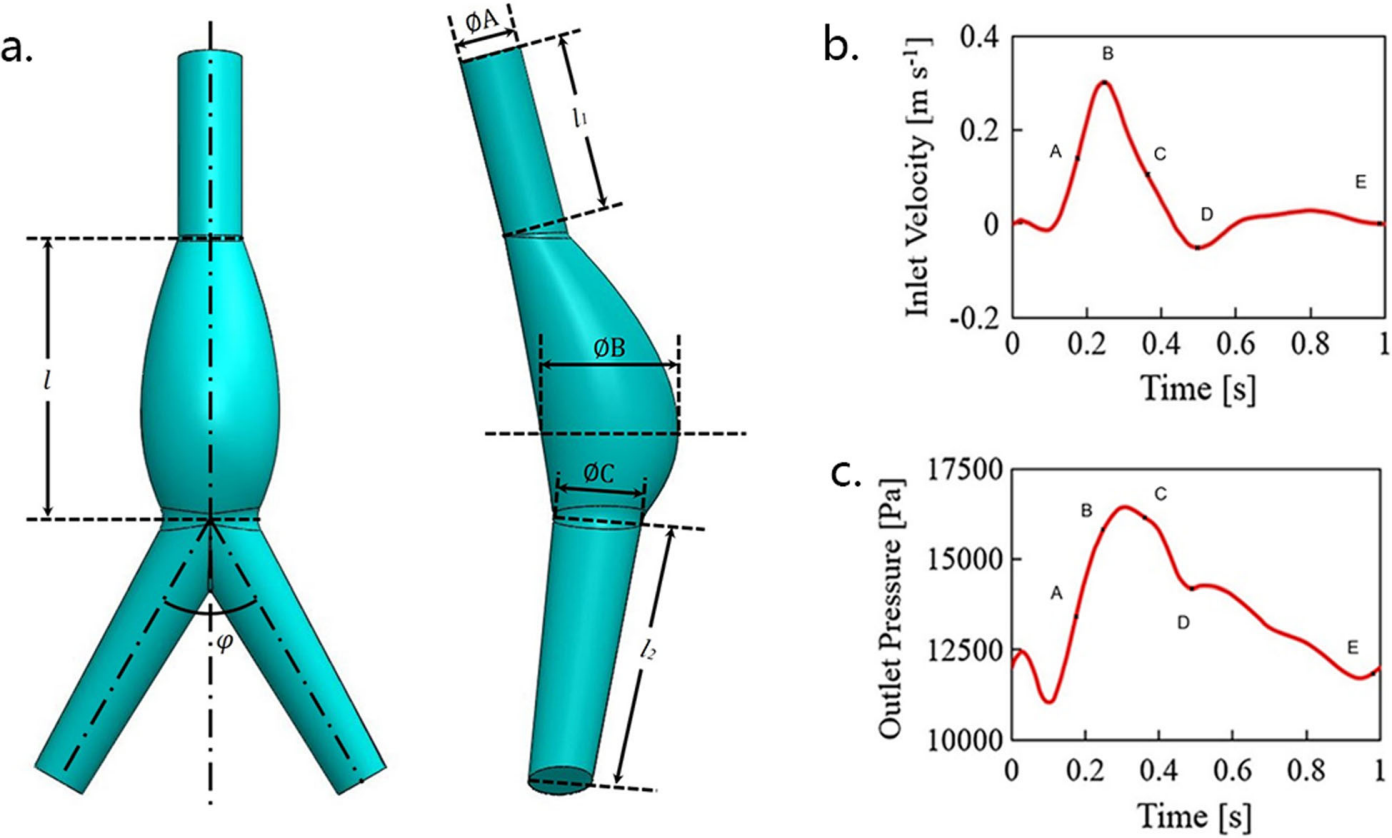
Uniformed model and boundary conditions. **a** Uniformed model of AAA for simulation (*l*, the length of the abdominal artery; *l*_*1*_, the length of the infra-renal artery; *l*_*2*_, the length of the common iliac artery; *φ*, the angle of iliac bifurcation; ∅A, infra-renal artery diameters; ∅B, maximum diameters; ∅C, common iliac artery diameters). **b** Inlet aortic velocity. **c** Outlet iliac pressure (five points from one cardiac cycle, early systole (A), peak systole (B), late systole (C), reflux (D), and end systole (E))

Since each AAA case model has its specific geometry, in order to analyze the common hemodynamic performances of each gender, the unified model of male and female AAAs were constructed based on the average of all male and female morphological parameters (Table 2) shown in Fig. 3a. In addition, the iliac bifurcation angle (*φ*) was chosen to be 60° as which was confirmed having the least effect by Xenos et al. [21]. All the parameters of the two uniform models are presented in Table 2 and Fig. 3a.

**Table 2:** Model parameters

Parameters	Female	Male
*l* (mm)	99.5	106.2
*l*_1_ (mm)	61.47	72.84
*l*_2_ (mm)	55.8	71.34
*φ* (°)	60	60
∅A (mm)	20.49	24.28
∅B (mm)	48.35	52.50
∅C (mm)	18.60	23.78

The specific values of each size of the models

*l* the length of the abdominal artery, *l*_*1*_ the length of the infra-renal artery, *l*_*2*_ the length of the common iliac artery, *φ* the angle of iliac bifurcation, *∅A* infra-renal artery diameters, *∅B* maximum diameters, *∅C* common iliac artery diameters

### Hemodynamic simulation

1. Assumptions and governing equations

In this study, the blood was assumed to be incompressible, homogeneous, and Newtonian fluid, and the fluid domain was governed by the Navier-Stokes equation and the continuity equation [22]:

6 $$ \uprho \left[\frac{\partial \overrightarrow{u}}{\partial t}+\left(u\bullet \nabla \right)u\right]+\nabla p-\mu {\nabla}^2\overrightarrow{u}=0 $$

7 $$ \nabla \bullet \overrightarrow{u}=0 $$

where $\overrightarrow{u}$ and *p* respectively represent fluid velocity vector and pressure. The density of blood (*ρ*) was 1050 kg ∙ m^−3^ with constant viscosity (μ) of 0.0035 Pa∙s [23, 24].The walls were considered to be rigid and no-slip. For continuity and velocity residuals, the convergence criterion was set to be 1 × 10^−5^.

2) Mesh generation

The ICEM (ANSYS, Inc., Canonsbury, PA, USA) software was used to generate meshes of the models, with a combination of hexahedral and tetrahedral elements. The total number of elements in the uniform model for male is 456,303 and for female is 444,293. The maximum and minimum dimensions of the mesh are 1.0 mm and 0.5 mm, the number of the boundary layer was set to 5, the height ratio was set to 1.1, and the total height was set to 0.61051 mm.

3) Boundary conditions

In order to study the performance of the models throughout the entire cardiac cycle, simulation calculations were carried out under pulsating blood flow conditions. The inlet velocity waveform and outlet pressure waveform are shown in Fig. 3b and c, from Olufsen et al. [25]. The pressure-velocity coupling was treated by the PISO [26] algorithm for the pulsatile flow. A second order upwind scheme [27] was employed for all convective diffusively transported variables. The simulations were performed for four cardiac cycles, with 200 steps in each cycle (*T* = 1 s). The time-averaged results are then obtained by averaging the values calculated during the last complete period.

### Data analysis

Statistical analysis was performed using the MATLAB programming environment (The Math-Works, Natick, Mass). The *χ*^2^ test was used to compare female and male groups, and a two-tailed *t* test was used for gender comparisons. The probability level of *P* < 0.05 was used to indicate statistical significance. The Tecplot software was used to calculate three wall shear stress (WSS)-based hemodynamic parameters for whole cardiac cycle. Their definition is based on a study by Chiastra et al. [28].

The time-averaged wall shear stress (TAWSS) was used to describe the features of WSS in pulsatile flow. The TAWSS was defined as follows:

8 $$ \mathrm{TAWSS}=\frac{1}{\mathrm{T}}{\int}_0^T\left|\mathrm{WSS}\left(s,t\right)\right|\bullet \mathrm{dt} $$

where *T* is the duration of the cardiac cycle and *s* is the position on the vessel wall.

The oscillatory shear stress index (OSI) on the inner wall of the models was calculated as:

9 $$ \mathrm{OSI}=0.5\left[1-\left(\frac{\left|\frac{1}{T}{\int}_0^T\mathrm{WSS}\left(s,t\right)\bullet \mathrm{dt}\right|}{\frac{1}{T}{\int}_0^T\left|\mathrm{WSS}\left(s,t\right)\right|\bullet \mathrm{dt}}\right)\right] $$

where WSS is a vector parameter and its direction changes with the cardiac cycle time. The OSI indicates the frequency of changes in the WSS direction, ranging from 0 (flow is one-directional without oscillations) to 0.5 (the WSS direction changes frequently).

The relative residence time (RRT) was calculated as:

10 $$ \mathrm{RRT}=\frac{1}{\left(1-2\bullet \mathrm{OSI}\right)\mathrm{TAWSS}} $$

RRT was used to determine the residence time of particles near the wall and recommended as single metric of low and oscillating shear stress. It is inversely proportional to the magnitude of the TAWSS vector and has obvious connections to the biological mechanisms of atherosclerosis.

## Results

### Morphological parameter comparisons

The morphological parameters are shown in Table 3. There was no significant difference in ASI (0.0293m^−1^ for females and 0.0291m^−1^ for males, *p* = 0.908) and the expansion factor (2.39 for females and 2.22 for males, *p* = 0.234).

**Table 3:** Morphological parameter comparisons

Gender	Female (*n* = 11)	Male (*n* = 23)	*p*
ASI (m^−1^)	0.0293 ± 0.0037	0.0291 ± 0.0061	0.908
Expansion factor	2.39 ± 0.347	2.22 ± 0.616	0.234
Lumbar vertebrae curve	0.01046 ± 0.00407	0.00748 ± 0.00240	0.0113
AAA centerlines	0.03809 ± 0.01713	0.02299 ± 0.00668	< 0.01
ILT volume (mm^2^)	22491.66 ± 15354.88	53091.35 ± 47030.15	0.044
ILT percentage volume	19.96% ± 14.90%	37.48% ± 21.14%	0.019

The volume of ILT and the percentage volume ILT of females were significantly smaller than males. The average ILT volume of females (22491.66 ± 15354.88 mm^3^) was smaller than that of males (53091.35 ± 47030.15 mm^3^) with *p* = 0.044. And the ILT percentage volume, which eliminated the effect of body size, also showed that females have less ILT (19.96% ± 14.90%) compared with males (37.48% ± 21.14%) with *p* = 0.019.

**Fig. 4:**
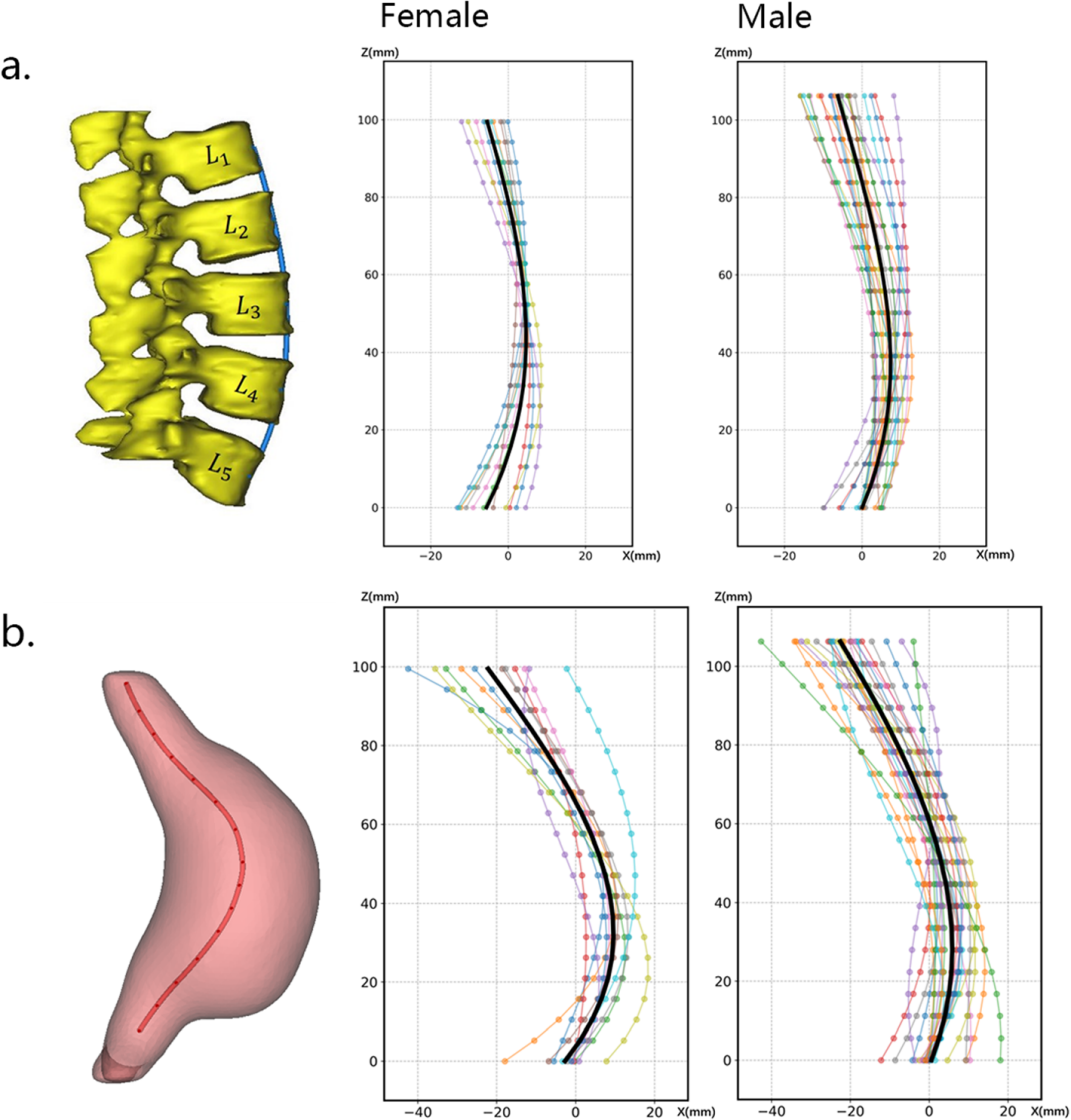
The mean lumbar vertebrae curves and mean centerlines of AAA composed from various curves projected to the sagittal plane of all male (*n* = 23) and female (*n* = 11) cases. **a** The average lumbar vertebrae curves (black line). **b** The average AAA centerlines curves (black line)

The averaged curvature of lumbar vertebrae curve and AAA centerline from female models was obviously higher than that of male. The results showed that female lumbar vertebrae has higher curvature (0.01046 ± 0.00407) than that of male (0.00748 ± 0.00240) with *p* = 0.0113, shown in Fig. 4a, which was consistent with the study of Hay et al. [29]. The AAA centerlines of each case and the composite AAA curves (black lines) are shown in Fig. 4b. The curvature of the centerlines was 0.03809 ± 0.01713 for female and 0.02299 ± 0.00668 for male with *p* = 0.000753.

### Hemodynamic simulation result

**Fig. 5:**
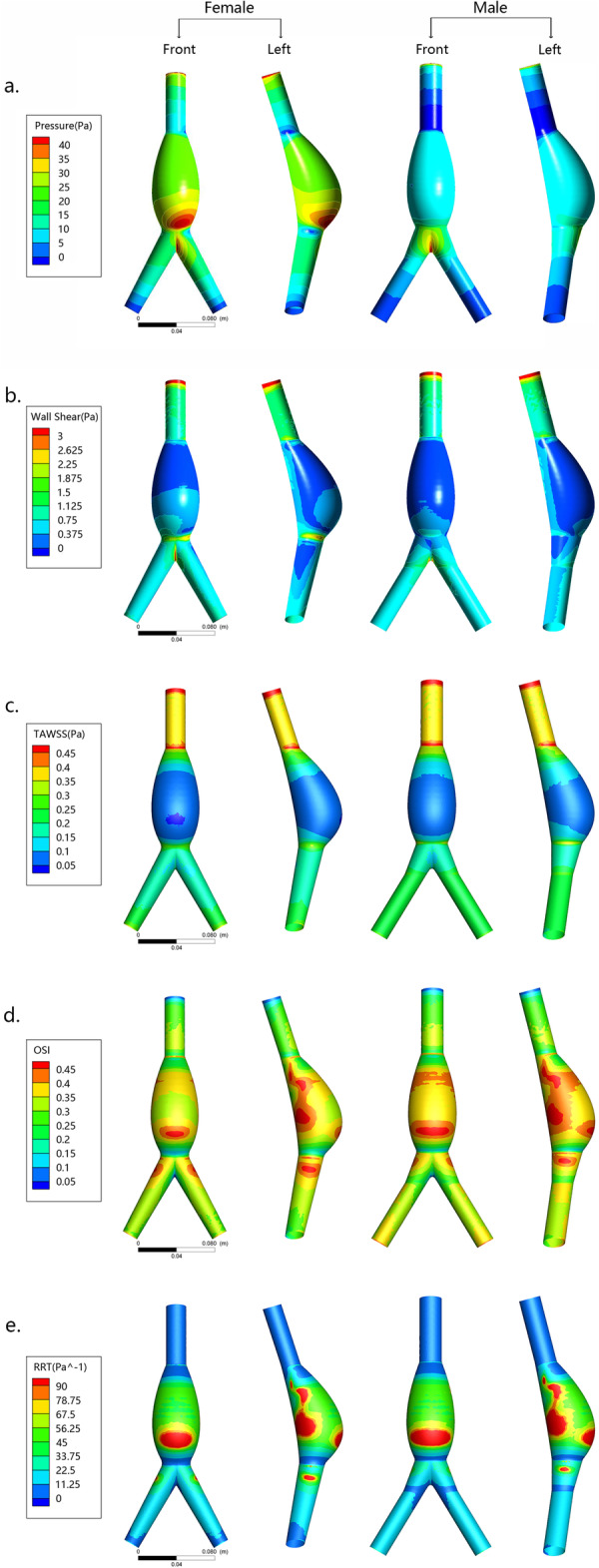
Distribution of hemodynamic parameters. **a** Pressure in inlet velocity peak (*v* = 0.33 m/s) point. **b** Wall shear stress in inlet velocity peak (*v* = 0.33 m/s) point. **c** Wall shear stress throughout the period, time-averaged wall shear stress (TAWSS). **d** The distribution of the oscillatory shear stress index (OSI). **e** The distribution of the relative residence time (RRT)

Figure 5 a shows the distribution of pressure on the wall at the peak systole time (*v* = 0.33 m/s, point B in Fig. 3b), and the magnitude of the pressure is indicated by color. The distributions of pressure were similar between male and female, and the peak pressure was found on the low anterior areas of both AAA models. However, the peak pressure of the female model was much higher than that of the male model (female vs. male = 61.4 vs. 46.3 Pa).

Figure 5b shows the distribution of the wall shear stress (WSS) at the peak systole time (point B). The contours indicate that male AAA model has a lower overall WSS compared to the female AAA, especially at the lower anterior area of the AAA. And the proportion of the area where the WSS of female AAA is less than 0.3 Pa is 27.069% and the WSS of male AAA is less than 0.3 Pa is 30.065%.

For the time-averaged WSS (TAWSS) distribution, similar observations were made as for the above-described WSS distribution. Figure 5c shows that the female model undertakes a smaller TAWSS during the entire cycle, but the difference is very small.

Figure 5d shows the distribution of the OSI on gender models. Relatively high OSI aggregations are found on the low anterior and back areas of AAA models. The OSI on the wall of male AAA model (0.333 ± 0.0782) was generally higher than that on the female AAA model (0.302 ± 0.0782).

The contours of RRT for both genders as shown in Fig. 5e present the same features as the OSI distribution. The higher RRT mainly distributes at the lower anterior and back areas of both AAA models, and the RRT on the male AAA model was relatively higher than that on the female AAA model. As shown in Fig. 5e, the proportion of the red part (RRT > 90Pa^−1^) of the female AAA model is less than that of males (female vs male = 4.93%: 7.52%).

## Discussion

Recent literatures have focused on the gender differences of cardiovascular diseases (CVDs) which initiates to raise awareness in research, clinical treatment, pharmacological development, device design, etc. for cardiovascular diseases [30]. So far, the uncovered reasons that may contribute to CVD gender differences are mainly related to different life habits and biological aspects, such as smoking, diabetes, hypertension, low/high density lipoprotein (LDL)-cholesterol levels, menopause, and hormone [12, 15, 31]. In the present study, morphological and hemodynamic differences between different genders were revealed by analyzing two groups of different gender AAA cases. These findings may provide new clues to the gender differences in CVDs.

Morphological features are very important intuitive indicators of AAA risk. Currently, the maximum diameter is widely used as the criteria for EVAR surgery. In this study, we found that the female AAA model has more complex geometries (smaller diameters, larger ratio of max to normal diameter, shorter centerline, more curved centerline and lumbar vertebrae curvature) than the male. The hemodynamic simulation based on gender-different models revealed that the female AAA models experienced a more complex and dangerous hemodynamic environment. The series of hemodynamic indicators shown in the results confirm the hypothesis that the morphological differences could induce different hemodynamic environments, thereby increasing the risk of rupture for female AAA.

Besides, this study found that in the unified model of male AAA, low WSSs, high OSIs, and high RRTs are more likely to appear, which are indicators of ILT formation [32]. Our statistical results of thrombus volume also confirmed this speculation. Since the ILT was usually regarded as a mechanical cushion that can reduce AAA wall stress by a shielding effect and allow AAA to tolerate greater stress without rupture [33–36], it further proves that the hemodynamics in male AAA models is apt to keep the stability of AAA.

The hemodynamic characteristics of artery are obviously influenced by the geometry of the arteries. The abovementioned hemodynamic differences between the female and male models are likely to be formed by morphological differences (especially the different curvature of AAA centerlines). The reason for AAA centerline curvature difference may be explained anatomically. The abdominal aorta is anatomically adjacent to the lumbar vertebrae, which has already been confirmed here and documented by previous studies: the lumbar vertebrae curvature of female is higher than that of male [29]. The curvature of AAA centerline can generate asymmetry of the blood lumen, thereby increasing the nonuniform stress distribution and the risk of rupture.

It was widely reported that females with AAA less often meet the current anatomical criteria (maximum diameter > 5 cm) for endovascular repair and experience worse perioperative and long-term survival [37]. Therefore, it is necessary to consider gender differences when evaluating AAA risk and seek more AAA indicators in addition to the current surgical criteria. According to current study, more morphological parameters besides the maximum diameter and hemodynamic factors are suggested to be involved.

It should be kept in mind that the CVDs are caused by multiple factors, like lifestyle, environment, gene, and hemodynamics, and so does the gender-difference of CVDs. Each factor could play roles together with one or several other factors. The present study added two new factors (morphology and hemodynamics) to this complicated problem, and their weights and exact roles in the entire story require more research to figure out.

As a preliminary study, there are still some shortcomings in this study. In this study, 11 female patients and 23 male patients were randomly selected as subjects of this study. Due to the patient specificity, greater numbers of patients are needed to be involved in the future study. In computational fluid dynamic (CFD) simulations, the AAA wall was considered to be rigid. This assumption may influence the magnitude but should not significantly affect the distribution of pressure and WSS [38]. Therefore, it should not affect the overall findings of our research.

## Perspectives and significance

Gender differences in cardiovascular disease have received widespread attention, and studies of gender differences in AAA rupture risk are critical to the treatment of AAA patients and to reduce mortality of them. In this study, morphological and hemodynamic differences between genders were revealed by analyzing different gender AAA models, which may provide new clues to gender differences in cardiovascular disease. This paper points out that it is necessary to consider gender differences when assessing AAA risk and seek more AAA indicators in addition to the current surgical criteria (such as more morphological parameters besides the maximum diameter and hemodynamics factors).

## Conclusions

The present study found that both the morphological and hemodynamic features have very obvious gender differences. The lumen of female AAA has smaller size (length and diameters), greater curvature, higher expansion ratio, and lower ILT volume. Female AAA models are subject to higher wall pressure, higher wall shear stress, and lower OSI (oscillatory shear). These morphological and hemodynamic indicators are likely to contribute to the higher rupture risk of AAA for female.

These findings would help to explore the mechanism of AAA gender differences together with other biological differences, and the conclusions draw more attention to gender-specific consideration of the treatment of AAA. More morphological and hemodynamic indictors are suggested to be involved in the future guidelines.

## Data Availability

Not applicable.
